# Radiomics-based models to predict IDH mutation status and prognosis in gliomas using MRI: a multicenter study

**DOI:** 10.3389/fonc.2026.1775958

**Published:** 2026-03-27

**Authors:** Esra Sümer-Arpak, Ayca Ersen Danyeli, M. Cengiz Yakicier, M. Necmettin Pamir, Koray Özduman, Alp Dinçer, Esin Ozturk-Isik

**Affiliations:** 1 Division of Embedded Intelligent Systems LAB, Department of Computer Science, Electrical and Space Engineering, Luleå University of Technology, Luleå, Sweden; 2 Institute of Biomedical Engineering, Boğaziçi University, Istanbul, Türkiye; 3 Department of Medical Pathology, Acibadem University, Istanbul, Türkiye; 4 Brain Tumor Research Group, Acibadem University, Istanbul, Türkiye; 5 Bahrain Oncology Center, King Hamad University Hospital, Muharraq, Bahrain; 6 Department of Neurosurgery, Acibadem University, Istanbul, Türkiye; 7 Department of Radiology, Acibadem University, Istanbul, Türkiye; 8 Center for Targeted Therapy Technologies, Boğaziçi University, Istanbul, Türkiye

**Keywords:** glioma, IDH mutation, machine learning, MRI, radiomics, survival

## Abstract

**Introduction:**

Gliomas are infiltrative primary intracranial tumors with marked biological and clinical heterogeneity. Prognosis varies widely and depends on tumor grade, histopathological characteristics, and molecular alterations. Isocitrate dehydrogenase mutation is a key prognostic biomarker and is associated with improved treatment response and longer overall survival. Radiomics enables the extraction of quantitative features from routinely acquired medical images. This study evaluated radiomics-based machine learning models for noninvasive prediction of isocitrate dehydrogenase mutation status and overall survival in glioma patients.

**Methods:**

From T2-weighted MRI scans of 638 gliomas (213 from a local institution (discovery), 425 from a public dataset (validation)), 1,820 radiomics features were extracted. Machine learning models were constructed and trained on the discovery cohort and externally validated to predict isocitrate dehydrogenase mutation status. A radiomics risk score was computed using Lasso regression, and patients were stratified into high- and low-risk groups using the median radiomics risk score for Kaplan-Meier analysis. Cox regression assessed the prognostic value of radiomics risk score along with clinical features (age, sex, WHO grade, isocitrate dehydrogenase mutation status). A nomogram incorporating independent predictors to estimate 1-, 2-, and 3-year overall survival was assessed using the concordance index and calibration curves.

**Results:**

Logistic regression and random forest classifier models achieved area under the receiver operating characteristic curve of 0.90 and 0.68 in the discovery and validation cohorts, respectively, for isocitrate dehydrogenase mutation prediction using 12 top radiomic features. High-risk patients showed significantly shorter median overall survival than low-risk patients in both discovery and validation cohorts (21 vs. 30 months, 10 vs. 19.5 months, respectively; P <0.001). Age, radiomics risk score, and isocitrate dehydrogenase mutation status were significant prognostic factors (P <0.05). The nomogram achieved concordance indices of 0.83 and 0.75 in the discovery and validation cohorts, with good calibration.

**Conclusion:**

Radiomics from preoperative T2-weighted MRI enabled prediction of isocitrate dehydrogenase mutation status and overall survival in gliomas. The radiomics risk score was an independent prognostic factor and, combined with clinical variables, enabled personalized risk stratification in gliomas. External validation further confirmed the generalizability of the proposed models.

## 1 Introduction

Gliomas account for approximately 25% of central nervous system (CNS) tumors and 81% of malignant brain tumors, with 5-year survival ranging from 5% to 95% depending on subtype, molecular features, grade, and other factors (1, 2). Isocitrate dehydrogenase (IDH) mutation is a key prognostic factor, associated with better overall survival (OS) and treatment response (3, 4). Although the IDH mutation has been detected by immunochemistry and genomic sequencing, intra-tumoral heterogeneity of gliomas might lead to incorrect results (5).

MRI plays a key role in glioma biology, prognosis, and outcome prediction. Various imaging features (e.g., location, contrast enhancement, edema) have correlated with IDH status and prognosis (6–11). Advanced MRI techniques, including perfusion and MR spectroscopy, have shown potential for estimating IDH status (12, 13) and prognosis (14, 15), thereby enhancing diagnostic and prognostic specificity (16). Identifying imaging markers that provide noninvasive information regarding IDH mutation status and survival is important, enabling appropriate treatment administration and prognosis estimation for gliomas.

The advancements in MR image analysis have enabled the transformation of the images into quantifiable representations. Radiomics extracts a large number of quantitative and mineable features from standard imaging modalities (17). Fully automated extraction of radiomics features from tumor segmentation results in three major feature categories: first-order intensity, shape, and texture features (17). Previous studies have demonstrated that radiomics has the ability to detect the IDH mutation status (18–21) and estimate the survival (20, 22) of gliomas from various MRI modalities. However, it is of utmost importance to externally validate the radiomics-based models to show the generalizability of the predictive power. Radiomics-based models often lack high predictive performance on unseen external data caused by differences in MRI protocol, MRI vendors, and feature extraction parameters (23). Recent works have shown improved generalizability of radiomics-based models by applying intensity normalization to MR images (21).

We hypothesized that quantitative radiomic parameters derived from MRI could represent glioma biology related to IDH mutational status and prognosis. Our study evaluated the predictive power of radiomics for the noninvasive preoperative estimation of IDH mutation status and prognosis in gliomas. Additionally, we also established a radiomics risk score (RS) and nomogram analysis for identifying patients who had high-risk profiles associated with poorer prognosis and reduced OS. We used a dataset from a local institution for model development, training, and testing for our analyses. Moreover, we performed external testing using the publicly available University of California San Francisco Preoperative Diffuse Glioma MRI (UCSF-PDGM) dataset.

## 2 Methods

### 2.1 Patients, imaging protocol, and tumor segmentation

In this retrospective study, two independent cohorts of 638 primary glioma patients with T2-weighted (T2w) MRI data were included. Patients were excluded based on the following criteria: (i) prior surgery or biopsy before imaging, (ii) absence of a histopathological diagnosis of primary glioma, and (iii) presence of multifocal or multicentric brain tumors.

The pre-operative imaging data of the discovery cohort were retrospectively collected from Acibadem University Altunizade Hospital after approval of the local ethics committee (Acibadem Healthcare Institutions Medical Research Ethics Committee (ATADEK-2016-9/10)). The discovery cohort consisted of 213 patients, who were scanned with a preoperative brain tumor MRI protocol, including T2w MRI (TR/TE:3470/107 ms, image size= 728×896×20, and pixel dimensions= 0.26×0.26×6.5 mm) at a 3 T clinical MR scanner (Siemens, Germany). Tumor segmentations were performed manually on T2w MRI using 3D Slicer and validated by a radiologist (24).

As the validation cohort, the publicly available UCSF-PDGM dataset from The Cancer Imaging Archive (TCIA) was used, which consisted of 425 gliomas (25). The validation cohort patients were scanned with a pre-operative brain tumor MRI protocol including T2w MRI (image size=240×240×155 and pixel dimensions= 1×1×1 mm) on a 3 T scanner (GE Healthcare, USA). Multicompartment tumor segmentations were provided for the validation cohort and merged into a single hyperintense tumor region on T2w MRI.

### 2.2 Pathological and genetic evaluation

The histopathological examinations of the discovery cohort were conducted on the surgically resected tissue to determine the tumor grade based on 2021 WHO CNS5 (26). Resected tissue samples were also investigated for IDH1/IDH2 mutations using minisequencing or Sanger sequencing methods (27). The IDH mutation status of the validation dataset (UCSF-PDGM) was obtained from TCIA, with details of the genetic tests described in the corresponding article (25).

### 2.3 Data preprocessing

**Figure 1:**
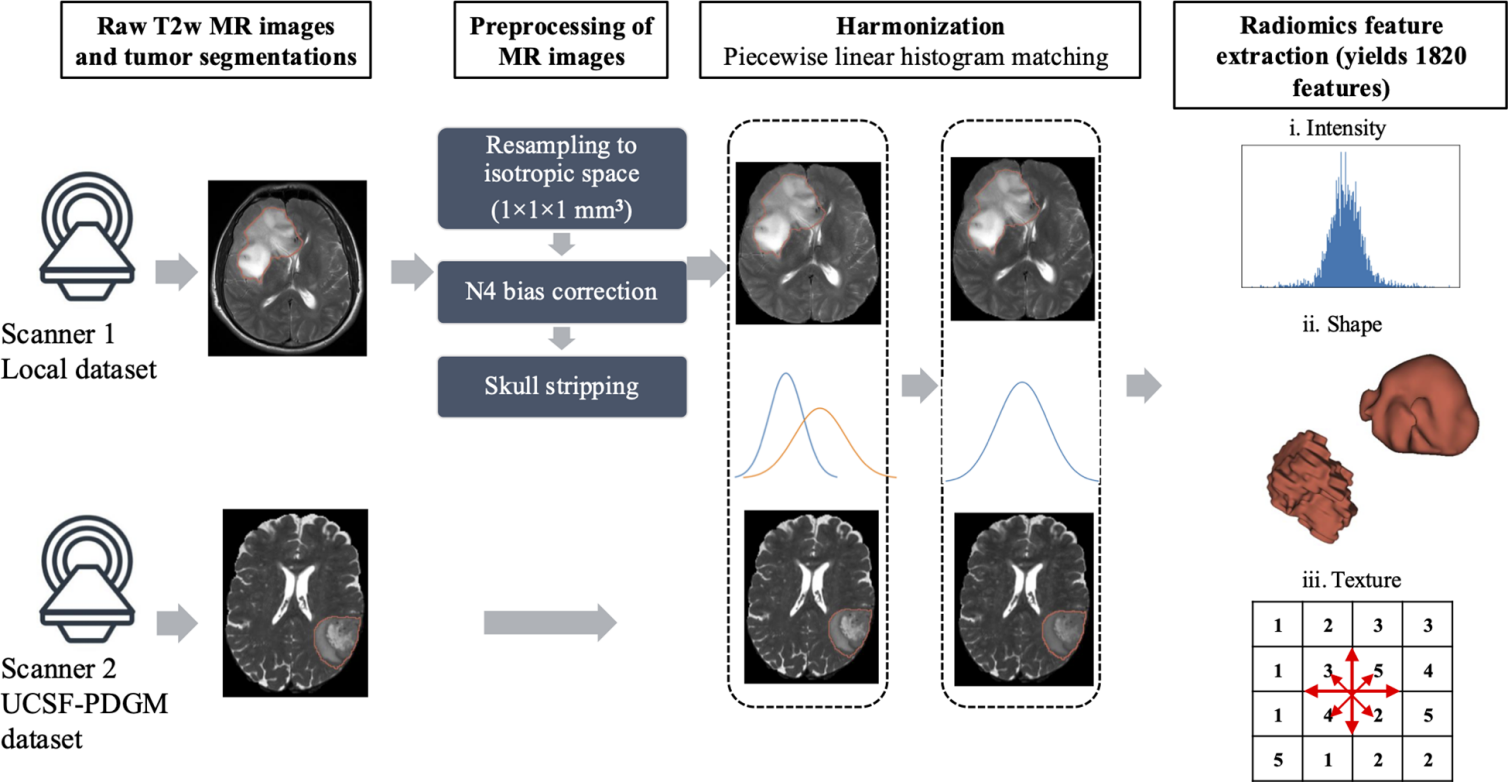
Workflow for image preprocessing and radiomics feature extraction.

To minimize the effect of any heterogeneity arising from different scanners and inter-subject variations, the images were preprocessed prior to feature extraction (**Figure 1**). For the discovery cohort, T2w MRI and corresponding tumor masks were resampled to isotropic voxel dimensions of 1×1×1 mm^3^. N4 bias correction was applied to correct spatial intensity heterogeneity on T2w MRI (28) followed by skull-stripping (29). The validation cohort images had an isotropic voxel dimension of 1×1×1 mm^3^ and underwent N4 bias correction and skull stripping. For the harmonization of two independent datasets, a piecewise linear histogram matching technique was applied to the preprocessed images (30).

### 2.4 Feature extraction, model construction, and external validation

**Figure 2:**
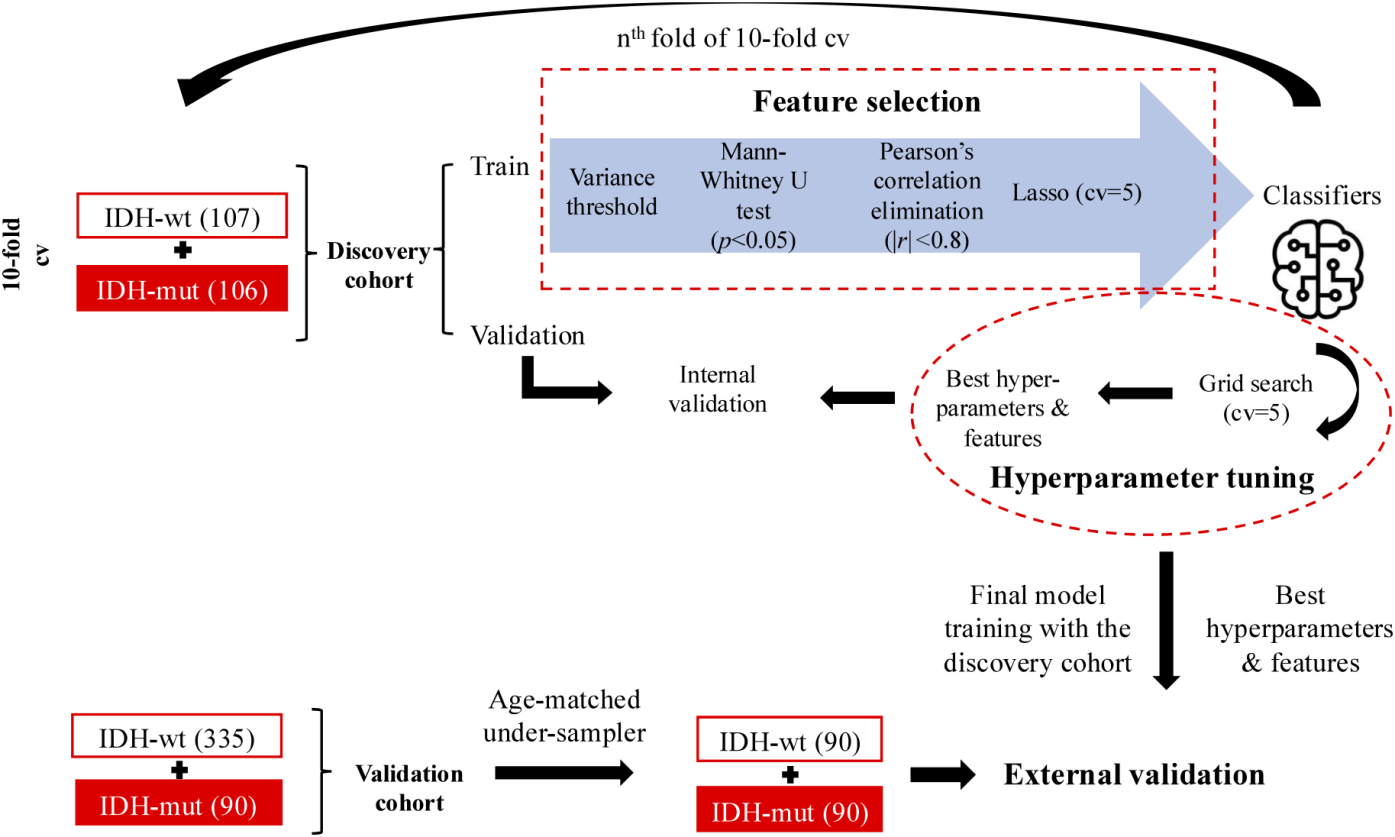
The overall pipeline of radiomics models using the discovery dataset is shown, with the models being externally validated using the validation cohort.

**Figure 2** shows the overall pipeline of IDH mutation estimation using radiomics-based machine learning models. 1,820 radiomic features, which included intensity, shape, and texture features, were extracted from discovery and validation cohorts. Detailed radiomic feature extraction settings are provided in **Supplementary Table 1**. The discovery cohort was used for feature selection, hyperparameter tuning, and model construction. More specifically, in the 10-fold cross-validation scheme, we first performed a feature selection procedure employing both unsupervised and supervised machine learning approaches on the training data of the discovery cohort. The final feature subset was determined with a selection frequency of ≥ 70% to avoid overfitting. The feature subset was then used as input for selecting the best hyperparameters of machine learning classifiers (k-nearest neighbor (kNN), logistic regression, support vector machines (SVM), linear discriminant analysis, decision trees, random forest, and extreme gradient boost (XGBoost)). A 5-fold cross-validated grid search was used to determine the hyperparameters. In the last step, we trained the tuned models with a complete set of samples of the discovery dataset. The IDH mutation of the discovery cohort was classified using the most selected features (n=12) with optimized hyperparameters, repeated 50×10-fold cross-validation. The validation cohort exhibited marked class imbalance, with IDH wildtype (IDH-wt) cases comprising 79% (n=335) of the cohort, compared with 21% (n=90) IDH mutant (IDH-mut) cases. To mitigate class imbalance problem while accounting for potential confounding due to age differences in the IDH-wt subgroups of discovery and validation cohorts, we performed distribution-matched undersampling based on age. Specifically, ages of IDH-wt subgroups in both cohorts discretized into equal width bins (n=10) spanning the observed age range of the discovery cohort. The age distribution of the validation group was computed across these bins, and participants were randomly sampled without replacement according to the discovery set proportions.

This procedure was repeated 50 times using different random seed values. The same feature subset selected in the discovery cohort was applied to the validation cohort. The predictive performance of the models was evaluated in the discovery and validation cohorts using accuracy, recall, precision, and area under the receiver operating characteristic curve (ROC AUC).

### 2.5 Radiomics-based risk score

The feature selection procedure was also applied to the discovery cohort to identify prognostically relevant features (**Figure 2**). Six features were selected: sphericity, maximum (with wavelet-LLH filtering), GLCM cluster prominence (with wavelet-HLH filtering), GLSZM zone variance (with wavelet-LLL filtering), skewness (3D LBP filtering), and GLZM small area low gray level emphasis (with square filtering). Detailed definitions of the selected radiomic features are provided in **Supplementary Table 2**. The RS for each patient was computed as the weighted sum of the selected feature values multiplied by their corresponding Lasso coefficients (RS = Σ feature value × Lasso coefficient). The derived RS model was then applied to both the discovery and validation cohorts.

### 2.6 Statistical and survival analyses

The selected radiomics features and age were compared between IDH-mut and IDH-wt gliomas using a Mann-Whitney U test with Bonferroni correction (P*<*0.0042). The sex distributions of the patients were compared between the IDH subgroups using a *χ*^2^ test.

Survival analyses were conducted for the discovery (alive: n = 138; dead: n = 53) and validation cohorts (alive: n = 213; dead: n = 211). Univariable Cox regression assessed the impact of RS and clinical features (age, sex, WHO grade, IDH status) on OS (P*<*0.05). Since IDH-wt tumors were classified as grade 4, which resulted in an overlap between IDH mutation status and tumor grade. To avoid non-discriminative estimates, multivariable Cox analyses were conducted with IDH mutation status, given its established role as a highly sensitive prognostic marker in adult diffuse gliomas (31). Patients in the discovery cohort were classified as high- or low-risk based on the median RS. This threshold was applied to the validation cohort, and Kaplan-Meier curves were compared using the log-rank test (P*<*0.05).

#### 2.6.1 Nomogram construction and validation

A nomogram was constructed using the independent risk factors identified in the multivariate Cox regression analyses to predict 1-year, 2-year, and 3-year OS in gliomas. The prognostic performance was evaluated on discovery and validation datasets using the concordance index (C-index). In addition, calibration curves were used to compare predicted and observed survival probabilities in 1-year, 2-year, and 3-year OS times with a 1000-bootstrap resampling strategy.

## 3 Results

### 3.1 Patient characteristics

**Table 1** summarizes the clinical and molecular-pathological characteristics of the patients. The median age was 45 years (range: 20–77) in the discovery cohort and 60 years (range: 19–94) in the validation cohort. While sex distributions were comparable between the discovery and validation cohorts (P = 0.60), patients in the validation cohort were significantly older (P = 5.67E-18). In the discovery cohort, 107 patients (50%) were IDH-mut, whereas in the validation cohort, 90 patients (21%) had IDH mutations. The sex distributions did not differ significantly between the IDH-mut and IDH-wt subgroups in either the discovery or validation cohorts (P = 0.42 and P = 0.65, respectively). However, patients in the IDH-wt subgroups were significantly older than those in the IDH-mut subgroups in both cohorts (P = 5.21E-17 and P = 1.63E-33, respectively).

**Table 1:** The patient characteristics for the discovery and validation cohorts.

Characteristic	Discovery cohort	Validation cohort
	(n = 213)	(n = 425)
Age median (range)	45 (20–77) years	60 (19–94) years
Sex (female)	81 (42%)	167 (39%)
IDH-wt	107 (50%)	335 (79%)
IDH-mut	106 (50%)	90 (21%)
WHO Grade 2	52 (24%)	42 (10%)
WHO Grade 3	50 (24%)	25 (6%)
WHO Grade 4	111 (52%)	358 (84%)
Oligodendroglioma (IDH-mut, 1p19q codeleted)	39 (18%)	13 (3%)
Astrocytoma (IDH-mut, 1p19q non-codeleted)	67 (32%)	77 (18%)
Glioblastoma (IDH-wt)	107 (50%)	335 (79%)

### 3.2 Identifying radiomic features associated with IDH mutation

**Figure 3:**
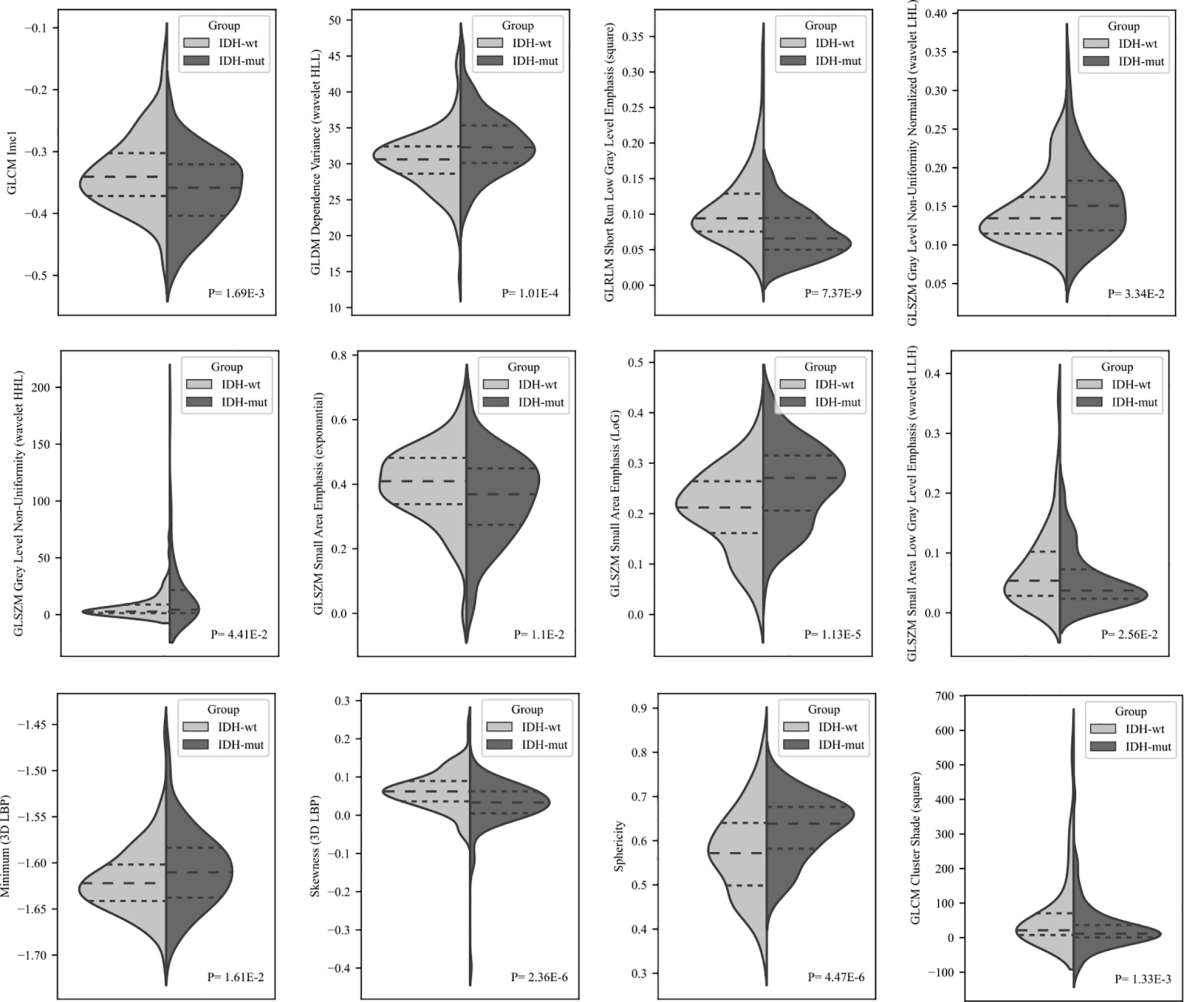
The violin plots show the distributions of 12 selected features in the IDH-wt and IDH-mut subgroups of the discovery cohort. For each pair of distributions, P-values were computed using a Mann-Whitney U test with Bonferroni correction (P*<*0.0042).

After the feature selection procedure, 12 radiomic features were retained with greater predictive value. The selected radiomic features and their definitions are summarized in **Supplementary Table 3**. The distributions of these 12 top-selected features in IDH-wt and IDH-mut gliomas of the discovery cohort are given in **Figure 3**. Seven radiomics features significantly differed between IDH-wt and IDH-mut gliomas in the discovery cohort (P*<*0.0042). IDH-wt tumors had significantly higher GLRLM short-run low gray-level emphasis (with square filtering), skewness (3D local binary pattern (LBP)), GLCM cluster shade (with square filtering), and GLCM Imc-1 (P*<*0.002, for all). On the other hand, IDH-mut tumors had significantly higher sphericity, GLSZM small area emphasis (with Laplacian of Gaussian filter (LoG)), and GLDM dependence variance (with wavelet high-low-low (HLL) filtering).

**Figure 4:**
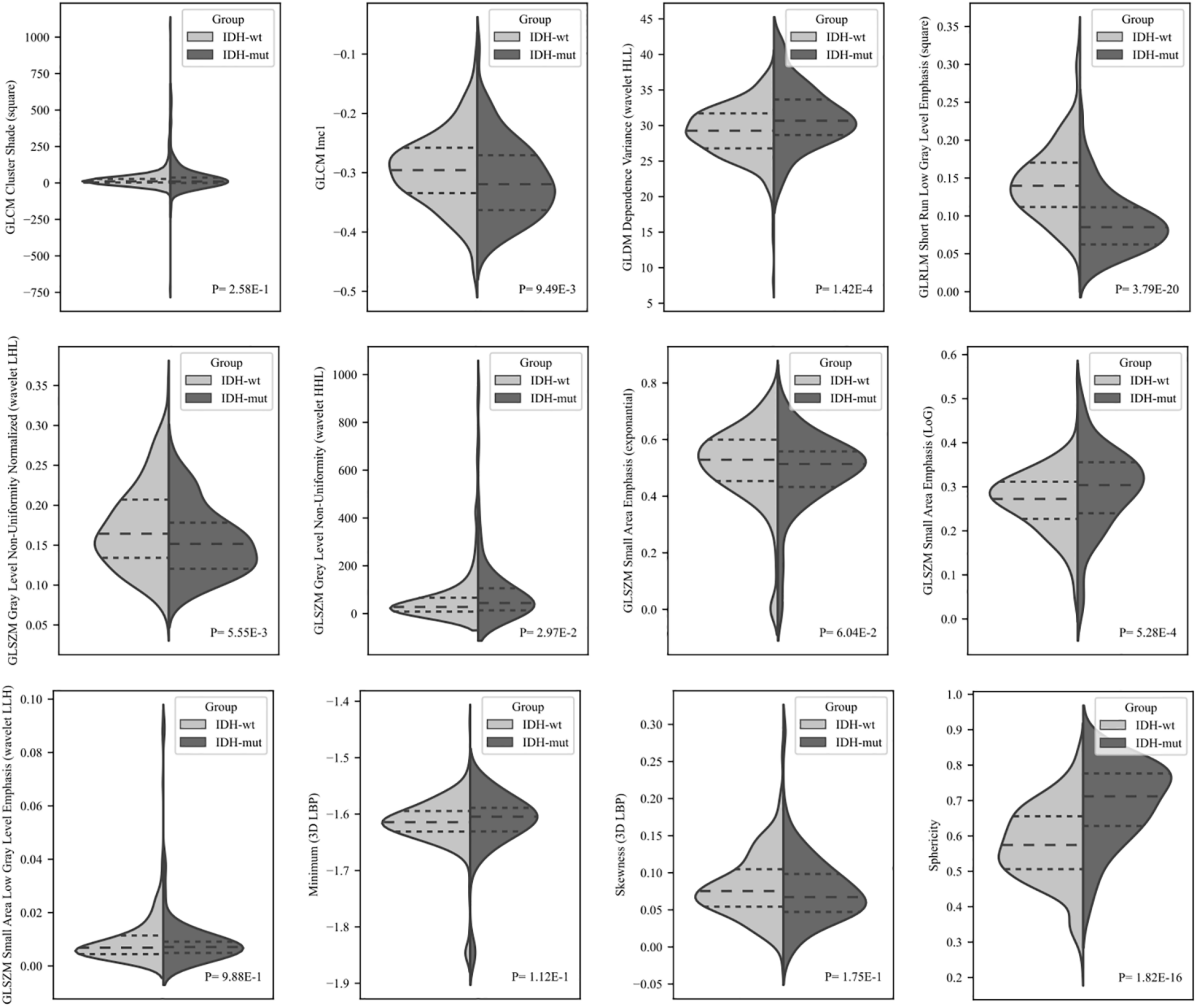
The differences in the top-selected radiomic features identified in the discovery cohort were further tested in the validation cohort using a Mann–Whitney U test with Bonferroni correction (P*<*0.0042) to discriminate between IDH-wt and IDH-mut tumors.

The same radiomics feature subset was used to identify differences between the IDH-mutational subgroups in the validation cohort, and their distributions are shown in **Figure 4**. Four of the selected radiomic features were significantly different between the IDH-wt and IDH-mut subgroups of the validation cohort (P*<*0.0042). While IDH-mut tumors had significantly higher sphericity, GLDM dependence variance (wavelet HLL filtering), and GLSZM small area emphasis (with LoG filtering), IDH-wt tumors showed significantly higher GLRLM short-run low gray-level emphasis (with square filtering) (P*<*0.001, for all).

### 3.3 Performance of radiomics classifiers in identifying IDH mutation

The predictive performances of the top three machine learning models for IDH mutation using radiomics features are presented in **Table 2**. In the discovery cohort, the best performance was obtained with logistic regression (accuracy = 0.80, recall = 0.78, precision = 0.83, ROC AUC = 0.90), followed by support vector machine (SVM) (accuracy = 0.80, recall = 0.79, precision = 0.81, ROC AUC = 0.90) and linear discriminant analysis (accuracy = 0.80, recall = 0.76, precision = 0.83, ROC AUC = 0.89).

**Table 2:** The classification performances of the top three radiomics-based machine learning models on discovery and validation cohorts.

50×10-fold cross-validation on the discovery cohort				
Model	Acc ± std	Recall ± std	Prec ± std	ROC AUC ± std
Logistic regression	0.80 ± 0.01	0.78 ± 0.01	0.83 ± 0.01	0.90 ± 0.01
SVM	0.80 ± 0.01	0.79 ± 0.01	0.81 ± 0.01	0.90 ± 0.01
Linear discriminant analysis	0.80 ± 0.01	0.76 ± 0.01	0.83 ± 0.01	0.89 ± 0.01
Validation cohort				
Random forest classifier	0.68 ± 0.02	0.72 ± 0.00	0.66 ± 0.02	0.68 ± 0.02
Decision tree classifier	0.66 ± 0.02	0.85 ± 0.00	0.62 ± 0.02	0.66 ± 0.02
Logistic regression classifier	0.65 ± 0.02	0.83 ± 0.00	0.61 ± 0.02	0.65 ± 0.02

In the validation cohort, IDH mutation was most effectively predicted using the random forest classifier (accuracy = 0.68, recall = 0.72, precision 0.66, ROC AUC = 0.68) and the decision tree classifier (accuracy = 0.66, recall = 0.85, precision = 0.62, ROC AUC = 0.66) classifiers (**Table 2**). In comparison, the logistic regression classifier achieved a slightly lower performance, with an accuracy of 0.65 (recall= 0.83, precision= 0.61, and ROC AUC = 0.65).

### 3.4 Performance of the survival prediction models

The median RS value of the discovery cohort (-0.0094) was used for risk stratification. The same threshold was subsequently applied to the validation cohort.

The median follow-up period was 24 months [range, 0–134] in the discovery cohort and 13 months [range, 0–89] in the validation cohort. In the discovery cohort, age distribution differed significantly between the low- and high-risk patient subgroups (P = 1.14E-8), whereas sex distribution was comparable (P = 0.06). Similarly, in the validation cohort, sex distribution did not differ significantly between the two risk subgroups (P = 0.29), while age distribution showed a significant difference (P = 1.63E-17).

**Figure 5:**
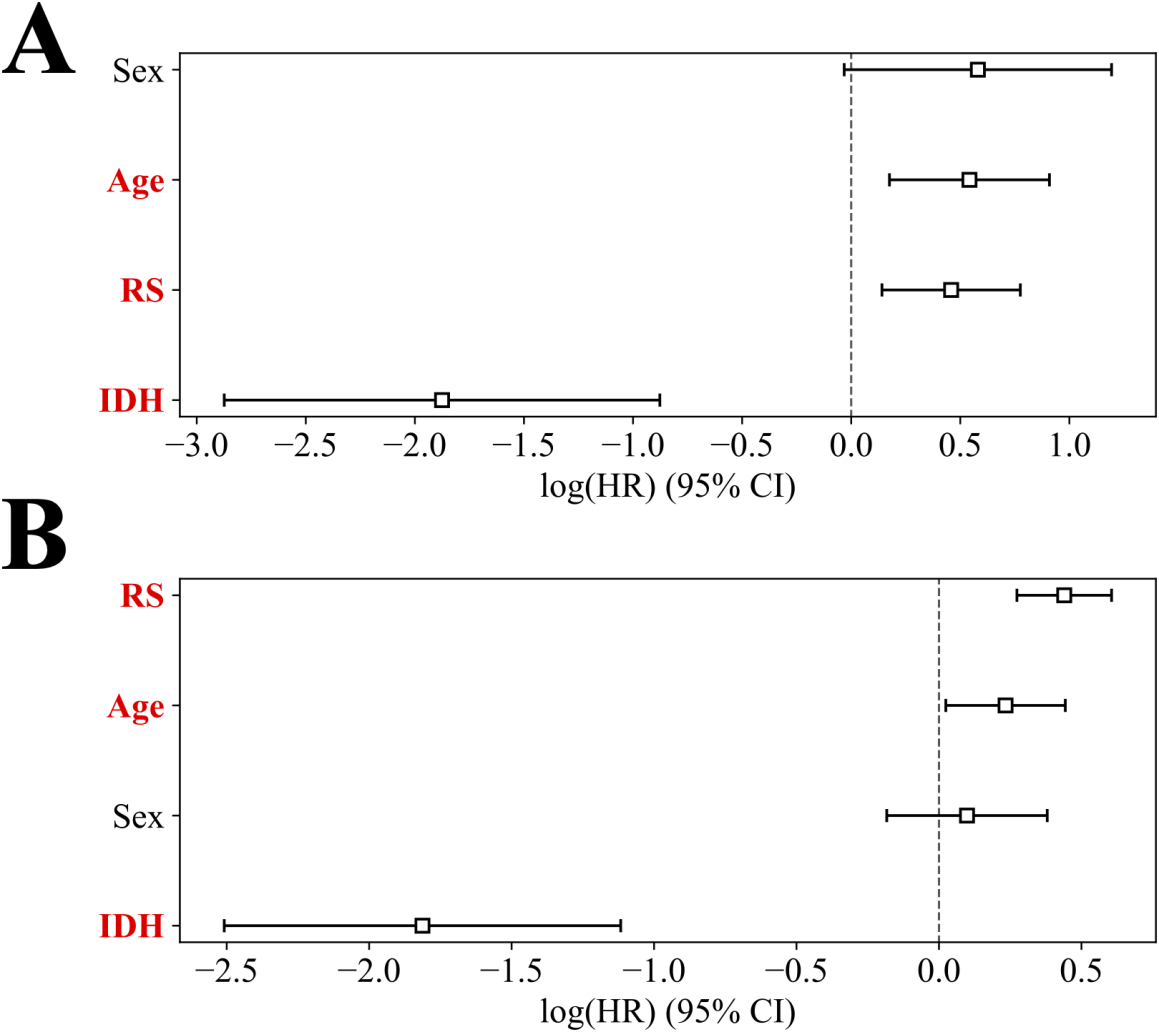
The impacts of RS, sex, age, and IDH mutation on OS in multivariable Cox analysis for the discovery cohort **(A)**, and the external validation cohort **(B)** (red: P*<* 0.05).

The results of the univariable and multivariable Cox proportional hazards models (hazard ratio (HR) and P values) are summarized in **Table 3**. Univariable Cox regression analysis of clinical features and RS revealed that age, RS, tumor grade, and IDH mutational status were significantly associated with OS in both the discovery and validation cohorts (P*<*0.005, for all). Multivariable Cox regression analyses confirmed that age, RS and IDH mutational status were independent prognostic factors for OS in the discovery cohort (P < 0.005, C-index = 0.84). Notably, age, RS, and IDH status were also associated with glioma OS in the validation cohort (P ≤ 0.03, C-index = 0.75). Furthermore, **Figure 5** shows the impacts of clinical features- age, sex, IDH status and RS on OS in multivariable Cox regression analysis (red: P*<*0.05).

**Table 3:** Univariable and multivariable Cox regression analysis for the discovery and validation cohorts.

Covariate	Univariable		Multivariable	
	HR (95% CI)	P	HR (95% CI)	P
Discovery cohort				
Age	2.95 (2.18, 3.99)	*<*0.005*	1.72 (1.19, 2.48)	*<*0.005*
Sex	1.79 (1.00, 3.18)	0.05	1.79 (0.97, 3.30)	0.06
RS	2.80 (2.17, 3.62)	<0.005*	1.58 (1.15, 2.17)	*<*0.005*
WHO Grade	7.17 (3.42, 15.03)	<0.005*	–	–
IDH	0.06 (0.02, 0.15)	<0.005*	0.15 (0.06, 0.42)	*<*0.005*
Validation cohort				
Age	2.18 (1.85, 2.57)	*<*0.005*	1.26 (1.02, 1.56)	0.03*
Sex	1.12 (0.85, 1.48)	0.42	1.10 (0.83, 1.46)	0.49
RS	2.01 (1.77, 2.29)	<0.005*	1.55 (1.31, 1.83)	<0.005*
WHO Grade	5.13 (2.95, 8.94)	<0.005*	–	–
IDH	0.09 (0.05, 0.16)	<0.005*	0.16 (0.08, 0.33)	<0.005*

"*" means P-value < 0.05.

**Figure 6:**
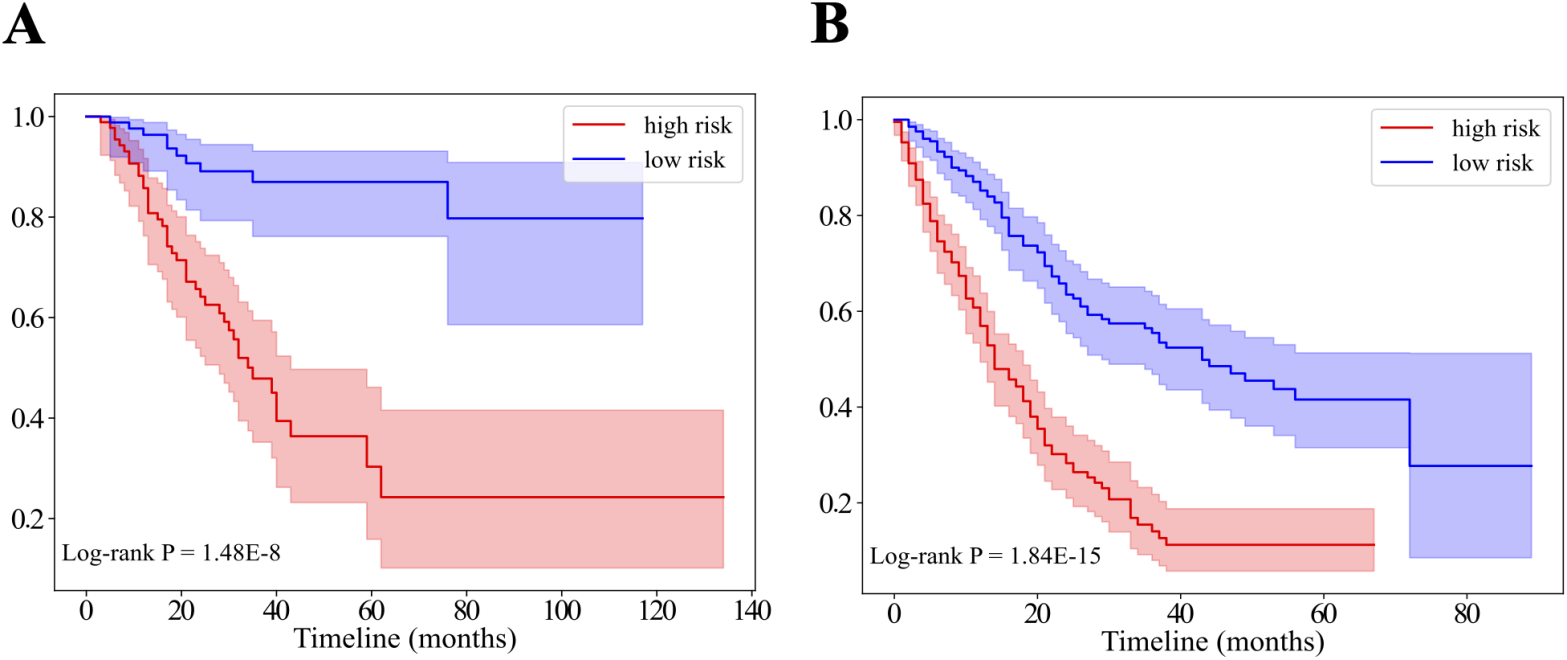
The Kaplan-Meier survival curves of the patients in the discovery **(A)** and the validation **(B)** cohorts are displayed as low-risk (blue) and high-risk (red) groups. The OS differences between the low and high-risk groups were assessed by log-rank tests.

Kaplan–Meier analysis showed that the RS stratified patients into high- and low-risk groups, with high-risk patients exhibiting significantly shorter median OS compared to low-risk patients in both the discovery (21 vs. 30 months, P = 1.48E-8) and validation (10 vs. 19.5 months, P = 1.84E-15) cohorts (**Figure 6**).

**Figure 7:**
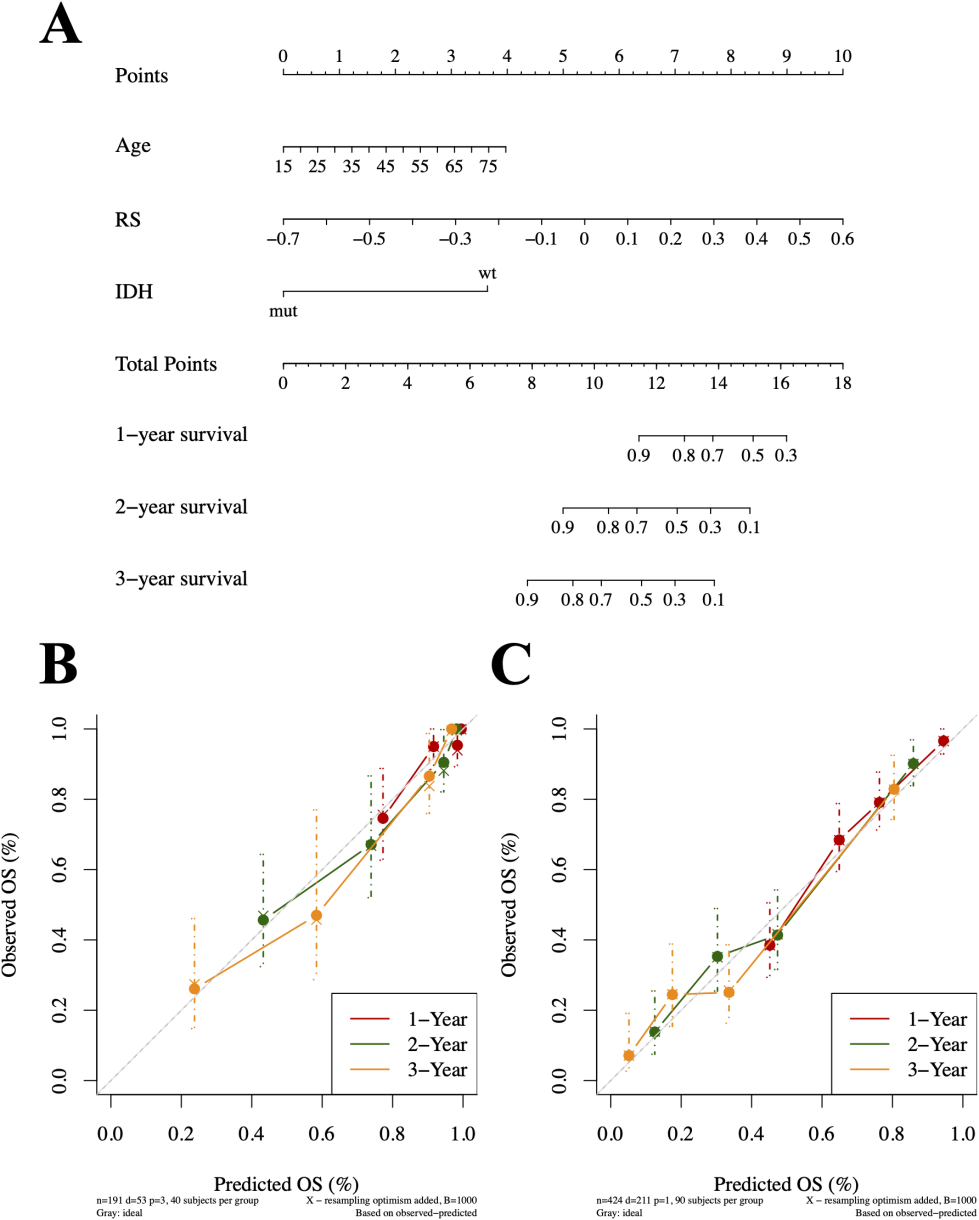
The nomogram for predicting 1-year, 2-year, and 3-year survival probabilities of glioma patients **(A)**. The nomogram integrated age, RS, and IDH status. The calibration curves for predicting 1-year (red line), 2-year (green line), and 3-year (orange line) survival probabilities of glioma patients in the discovery **(B)** and the validation cohorts **(C)**. The performances were estimated by bootstrapping 1000 repetitions.

The nomogram that integrated age, RS, and IDH status as predictors is shown in **Figure 7A**. The nomogram demonstrated that the RS score was the strongest contributor, followed by age, IDH status, and WHO tumor grade. It achieved good prognostic performance, with C-indices of 0.83 in the discovery cohort and 0.75 in the validation cohort. Calibration curves further indicated good agreement between predicted and observed survival probabilities, demonstrating high calibration accuracy in both datasets (**Figures 7B, C**).

## 4 Discussion

MRI is an essential imaging modality for the diagnosis and management of gliomas. In routine clinical practice, molecular subtyping and prognostic assessment rely on histopathological analysis of tumor tissue, which is invasive. Consequently, there is growing interest in noninvasively determining molecular subtypes from preoperative MRI (32). Radiomics has increasingly been utilized in gliomas to support precision medicine, yet the stability and generalizability of radiomics-based models remain major bottlenecks for clinical translation. To address these challenges, we developed radiomics-based models based on preoperative T2w MRI after applying a piecewise linear histogram matching technique, aiming to predict IDH mutation status and establish a prognostic model for gliomas. Importantly, the prediction models were evaluated on an independent external dataset. Our radiomics-based models demonstrated strong performance in predicting both IDH mutation status and patient prognosis.

A range of noninvasive MRI biomarkers, including necrosis, contrast enhancement, mass effect, perfusion on MRI have previously been investigated to predict IDH mutation and prognosis in gliomas (33). In addition, several visually assessed MRI signs have been associated with IDH mutation status and survival in gliomas, most notably the T2-FLAIR mismatch sign (34) and hyperintense FLAIR rim sign (35). The T2-FLAIR mismatch sign has been reported as a highly specific imaging biomarker for IDH-mut and 1p/19q noncodeleted gliomas (astrocytomas) with positive predictive value of 100% and associated with longer OS (36). Nevertheless, more recent studies have identified potential false-positive cases and limited sensitivity of this sign (37). Conversely, the hyperintense FLAIR rim sign has demonstrated higher sensitivity for presence of IDH mutation and 1p/19q noncodeletion, but its presence in grade 4 gliomas diminishes its specificity for molecular classification (38). Collectively, these limitations of MRI findings highlight the need for quantitative and reproducible imaging approaches beyond subjective visual interpretation. Radiomics offers such an approach by extracting high-dimensional quantitative features that capture the tumor heterogeneity beyond human visual perception. While previous studies have shown that combining radiomic features from multiple MRI modalities can improve predictive performance (19), we specifically focused on T2w MRI in the present study, as this sequence is routinely acquired, widely available across institutions, and less susceptible to protocol variability than advanced or contrast-dependent sequences. Notably, Bangalore Yogananda et al. (39) demonstrated that T2w MRI can predict IDH mutation status with performance comparable to that of multi-contrast MRI approaches. This observation is further supported by Zhang et al. (18), who reported that T2w-derived radiomic features exhibited stronger discriminative power for IDH mutation status than features extracted from T1-weighted or FLAIR images. Accordingly, we intentionally restricted our analysis to T2w MRI to maximize reproducibility and cross-center applicability. While multimodal MRI may yield higher performance, reliance on a single, standardized sequence facilitates more robust external validation and enhances the potential for broad clinical translation.

Radiomics features encompassing intensity, shape, and texture have been widely investigated for predicting IDH mutation status in gliomas. In our study, texture features demonstrated the greatest discriminative power, comprising nine of the 12 selected features. Among these, seven showed significant differences between the IDH subgroups in the discovery cohort, and four remained significant in the validation cohort. IDH-wt tumors exhibited lower sphericity, indicating more irregular boundaries and greater aggressiveness (40). Beyond tumor shape, intratumoral heterogeneity, particularly pronounced in IDH-wt tumors (glioblastomas), has been associated with poor prognosis (41). Previous studies have demonstrated that texture features correlate with intratumoral heterogeneity, capturing imaging patterns that may not be readily discernible to the naked eye (42). Texture features such as GLRLM short run low gray level emphasis captured necrotic, heterogeneous regions in IDH-wt tumors. GLSZM small area emphasis suggested uniform textures in IDH-wt tumors, possibly due to over-smoothing by Gaussian filtering. In contrast, GLCM Imc-1 approached -1 in IDH-mut tumors, indicating more homogeneous structures, while higher GLCM cluster shade in IDH-wt tumors reflected increased texture asymmetry. GLDM dependence variance, indicating pattern diversity, was higher in IDH-mut tumors, likely due to pronounced edema seen in T2w MRI. Additionally, higher skewness in IDH-wt tumors suggested asymmetric intensity distributions. These findings, partly consistent with previous studies (43–45), highlight the added value of texture analysis in identifying IDH mutation status beyond histogram and shape features.

Previous studies report IDH prediction ROC AUCs of 0.73–0.97 (18, 19, 43–48), often based on single-institution data. Our study advances this by using an external validation dataset, improving model generalizability. In the discovery cohort, radiomics-based models achieved good accuracy in cross-validated IDH mutation detection, with an ROC AUC of 0.90. However, in the validation cohort, the predictive performance declined, yielding an ROC AUC of 0.68. This reduction in predictive performance in the external validation cohort likely reflects differences in imaging protocols and scanner heterogeneity, which are well-known challenges in radiomics studies. It also highlights the potential for overfitting when models are trained and evaluated within a single cohort.

In addition to imaging-related differences, patient population heterogeneity between the discovery and validation cohorts may have contributed to the classification performance variation between two cohorts. Notably, the validation cohort exhibited class imbalance, with substantially fewer IDH-mut cases compared with IDH-wt cases. Furthermore, patients in the validation cohort were significantly older than those in the discovery cohort, and age differed between IDH-mut and IDH–wt subgroups in both cohorts. There has been an established association between age and development of more aggressive glioma subtypes (glioblastoma) (49). To mitigate the potential confounding effect of age and class imbalance issue, we performed age-matched undersampling in the validation cohort, ensuring comparable age distributions between IDH-wt subgroups across cohorts. Additionally, the survival model was developed using the discovery cohort, which included a broader short- and long-term survival spans, enabling RS to capture heterogeneous prognostic information. When applied to the IDH-wt dominant validation cohort with shorter follow-ups, the RS remained capable of correctly ranking patients according to risk. The preservation of risk stratification despite molecular imbalance supported the robustness and generalizability of the proposed radiomics-based prognostic model. Together, differences in age distribution, tumor aggressiveness, and follow-up duration emphasized the challenges of external validation across heterogeneous populations and the importance of evaluating radiomics models in diverse clinical settings to ensure robust generalizability.

Prior studies have identified multiple prognostic factors in gliomas; however, no consensus has been established regarding risk assessment (50). In our analysis, univariable Cox regression identified age, WHO tumor grade, and IDH status as independent prognostic factors for OS. In the multivariable model, clinical factors including age and IDH mutation status remained independently associated with OS. Tumor grade was not included in the multivariable analysis since it substantially overlapped with IDH mutation status, resulting in non-discriminative estimates. Additionally, histological tumor grading is subject to well-documented inter-rater variability, which may further limit its independent prognostic contribution (51). Overall, worse outcomes were associated with older age, higher tumor grade, and absence of IDH mutation, findings consistent with prior reports (4, 52).

Radiomics-based models have increasingly been recognized as independent prognostic tools (22, 53–55), providing complementary value to established clinical and molecular factors. In the present study, the RS was significantly associated with OS, with higher RS values corresponding to poorer outcomes. The nomogram integrating age, IDH mutation status, and RS demonstrated good prognostic performance and calibration, enabling individualized survival estimation. Notably, RS exhibited stronger prognostic contribution than individual clinical variables within the multivariable framework. These findings suggest that the proposed RS captures imaging phenotypes that are not exclusively driven by only IDH mutation status, but rather reflect complementary aspects of tumor biology relevant to patient outcome.

This study has several limitations. First, tumor segmentations in the discovery cohort were performed by a researcher under the supervision of a junior radiologist, and the assessment of inter-observer variability was not feasible. Manual and semi-automatic tumor delineations are subject to inter-observer variability, which can substantially influence radiomics feature values and model performance in the discovery cohort (56). Previous studies have shown that radiomics feature stability differs across feature categories, with shape features exhibiting the highest reproducibility, followed by first-order and GLCM features, whereas higher-order texture features tended to be more sensitive to segmentation variability (57). In the present study, the final feature subsets included features from categories reported to exhibit higher reproducibility, as well as higher-order texture features that are more sensitive to segmentation variability. Consequently, segmentation-related variability may have introduced additional variance in the radiomics features, underscoring the importance of standardized segmentation protocols to improve reproducibility and generalizability. The use of automated segmentation methods may further reduce observer-dependent variability and enhance reproducibility in radiomics studies (58). Second, while the present study focused on conventional T2w MRI to maximize clinical applicability and reproducibility, the exclusion of advanced MRI modalities limited the predictive power of models. Advanced imaging techniques, such as diffusion-weighted imaging and perfusion MRI, provide complementary information on tumor microstructure, vascularity, and cellularity that further enhance the prediction of IDH mutation status and patient prognosis (48, 59, 60). Therefore, future work should integrate multimodal radiomics frameworks that combine T2w MRI with advanced MRI sequences. Third, the validation cohort had fewer IDH-mut cases, requiring undersampling to match the class distribution. We selected to use age stratified undersampling strategy because other factors were homogeneous. Otherwise, one could use multivariate stratification methods or class-weighting models which allows retention of all available data. Lastly, although external validation was performed, further evaluation on larger, multi-institutional cohorts is necessary to confirm generalizability. In conclusion, radiomics from preoperative T2w MRI effectively predicted IDH mutation and OS in gliomas. Sphericity and texture features were key indicators, and RS was an independent prognostic factor. Integrated with clinical variables in a nomogram, RS improved personalized risk stratification. External validation confirmed models’ generalizability, supporting the integration of radiomics with clinical and molecular markers for precision medicine in gliomas.

## Data Availability

The raw data supporting the conclusions of this article will be made available by the authors, without undue reservation.
