# Supplementary material for: Radiomics-based models to predict IDH mutation status and prognosis in gliomas using MRI: a multicenter study
Source: Front Oncol. 2026 Mar 27;16:1775958. doi: 10.3389/fonc.2026.1775958 (PMC13065654; doi:10.3389/fonc.2026.1775958)
Supplement: Supplementary file 1 [file DataSheet1.pdf]

Supplementary Material

1 SUPPLEMENTARY TABLES

Feature definitions were obtained from the official PyRadiomics documentation van Griethuysen et al. (2017); PyRadiomics Development Team (2026).

Table S1. Radiomics feature-extraction settings and image types used in the study.

Category	Setting	Value
Image preprocessing	Resampling	$1\times1\times1$ mm^3
Image preprocessing	Interpolation	sitkBSpline
Image preprocessing	Intensity discretization	Fixed bin width
Image preprocessing	Bin width	32
Image type	Original image	Yes
Image type	Laplacian of Gaussian	$\sigma = [2.0, 3.0, 4.0, 5.0]$
Image type	Wavelet	Yes
Image type	Exponential	Yes
Image type	Gradient	Yes
Image type	3D local binary pattern	Radius = 1.0
Image type	Logarithm	Yes
Image type	Square	Yes
Image type	Square root	Yes
Feature classes	Shape	Yes
Feature classes	First-order statistics	Yes
Feature classes	Texture (GLCM)	Yes
Feature classes	Texture (GLRLM)	Yes
Feature classes	Texture (GLSZM)	Yes
Feature classes	Texture (GLDM)	Yes
Software	Radiomics library	PyRadiomics
Software	Version	v3.1.0

Table S2. Selected radiomic features for computation of radiomics risk score (RS), along with their corresponding image filters, feature classes, and definitions. Texture features were derived from the Gray Level Size Zone Matrix (GLSZM), Gray Level Run Length Matrix (GLRLM), Gray Level Co-occurrence Matrix (GLCM), and Gray Level Dependence Matrix (GLDM). Wavelet-based features (e.g., HLL: High-Low-Low frequency decomposition) and other filtered images (Laplacian of Gaussian (LoG), 3D local binary pattern (LBP-3D), exponential, square) were used PyRadiomics Development Team (2026)

Feature name	Image type	Feature class	Definition
Skewness	LBP-3D	First Order	Measures the asymmetry of the distribution of intensity values about the mean.
Small Area Low Gray Level Emphasis	Square	GLSZM	Measures the proportion of the joint distribution of smaller size zones with lower gray-level values in the image.
Sphericity	Original	Shape	Measures the roundness of the tumor region relative to a sphere. A value of 1 indicates a perfect sphere.
Maximum	Wavelet-LLH	First Order	The maximum gray-level intensity within the ROI.
Cluster Prominence	Wavelet-HLH	GLCM	Measures the skewness and asymmetry of the GLCM.
Zone Variance	Wavelet-LLL	GLSZM	Measures the variance in zone-size volumes within the GLSZM.

Table S3. Selected radiomic features included in the IDH mutation classification model, along with their corresponding image filters, feature classes, and definitions. Texture features were derived from the Gray Level Size Zone Matrix (GLSZM), Gray Level Run Length Matrix (GLRLM), Gray Level Co-occurrence Matrix (GLCM), and Gray Level Dependence Matrix (GLDM). Wavelet-based features (e.g., HLL: High-Low-Low frequency decomposition) and other filtered images (Laplacian of Gaussian (LoG), 3D local binary pattern (LBP-3D), exponential, square) were used.

Feature name	Image type	Feature class	Definition
Gray Level Non-Uniformity	Wavelet-HHH	GLSZM	Measures the variability of gray-level intensity values in the image, with lower values indicating greater homogeneity.
Short Run Low Gray Level Emphasis	Square	GLRLM	Measures the joint distribution of shorter run lengths with lower gray-level values.
Minimum	LBP-3D	First Order	The lowest voxel intensity value within the tumor region.
Sphericity	Original	Shape	Measures the roundness of the tumor region relative to a sphere. A value of 1 indicates a perfect sphere.
Small Area Emphasis	LoG (sigma = 4 mm), Exponential	GLSZM	Measures the distribution of small-size zones, with higher values indicating a greater proportion of small zones and finer textures.
Small Area Low Gray Level Emphasis	Wavelet-LLH	GLSZM	Measures the proportion of the joint distribution of small-size zones with lower gray-level values.
Gray Level Non-Uniformity Normalized	Wavelet-LHL	GLSZM	Measures the variability of gray-level intensity values normalized by the total number of zones, with lower values indicating greater similarity in intensity values.
Informational Measure of Correlation (Imc1)	Original	GLCM	Measures the correlation between the probability distributions of i and j using mutual information. A value of 0 indicates independence.
Skewness	LBP-3D-m1	First Order	Measures the asymmetry of the intensity value distribution about the mean.
Cluster Shade	Square	GLCM	Measures the skewness and uniformity of the GLCM. Higher values indicate greater asymmetry about the mean.
Dependence Variance	Wavelet-HLL	GLDM	Measures the variance in dependence size within the image.

REFERENCES

[Dataset] PyRadiomics Development Team (2026). Pyradiomics documentation

van Griethuysen, J. J. M., Fedorov, A., Parmar, C., Hosny, A., Aucoin, N., Narayan, V., et al. (2017). Computational radiomics system to decode the radiographic phenotype. *Cancer Res* 77, E104–E107. doi:10.1158/0008-5472.Can-17-0339
